# A fair machine learning model to predict flares of systemic lupus erythematosus

**DOI:** 10.1093/jamiaopen/ooaf072

**Published:** 2025-07-26

**Authors:** Yongqiu Li, Lixia Yao, Yao An Lee, Yu Huang, Peter A Merkel, Ernest Vina, Ya-Yun Yeh, Yujia Li, John M Allen, Jiang Bian, Jingchuan Guo

**Affiliations:** Department of Health Outcomes and Biomedical Informatics, University of Florida, Gainesville, FL, 32611, United States; Polygon Health Analytics LLC, Chalfont, PA, 18914, United States; Pharmaceutical Outcomes & Policy, University of Florida, Gainesville, FL, 32611, United States; Department of Biostatistics and Health Data Science, Indiana University School of Medicine, IN, 46202, United States; Center for Biomedical Informatics, Regenstrief Institute, Indianapolis, IN, 46202, United States; Division of Rheumatology, Department of Medicine, University of Pennsylvania, Philadelphia, PA, 19104, United States; Division of Epidemiology, Department of Biostatistics, Epidemiology, and Informatics, University of Pennsylvania, Philadelphia, PA, 19104, United States; Division of Rheumatology, College of Medicine, University of Arizona, Tucson, AZ, 85721, United States; Pharmaceutical Outcomes & Policy, University of Florida, Gainesville, FL, 32611, United States; School of Pharmacy, University of Maryland, Baltimore, MD, 21201, United States; Department of Pharmacy Practice, Purdue University College of Pharmacy, IN, 46202, United States; Department of Biostatistics and Health Data Science, Indiana University School of Medicine, IN, 46202, United States; Center for Biomedical Informatics, Regenstrief Institute, Indianapolis, IN, 46202, United States; Pharmaceutical Outcomes & Policy, University of Florida, Gainesville, FL, 32611, United States

**Keywords:** systemic lupus erythematosus, machine learning, prediction, fairness, social determinants of health

## Abstract

**Objective:**

Systemic lupus erythematosus (SLE) is a chronic autoimmune disease that disproportionately affects women and racial/ethnic minority groups. Predicting disease flares is essential for improving patient outcomes, yet few studies integrate both clinical and social determinants of health (SDoH). We therefore developed FLAME (**FLA**re **M**achine learning prediction of SL**E**), a machine learning pipeline that uses electronic health records (EHRs) and contextual-level SDoH to predict 3-month flare risk, emphasizing explainability and fairness.

**Materials and Methods:**

We conducted a retrospective cohort study of 28 433 patients with SLE from the University of Florida Health (2011-2022), linked to 675 contextual-level SDoH variables. We used XGBoost and logistic regression models to predict 3-month flare risk, evaluating model performance using the area under the receiver operating characteristic (AUROC). We applied SHapley Additive exPlanations (SHAP) values and causal structure learning to identify key predictors. Fairness was assessed using the equality of opportunity metric, measured by the false-negative rate across racial/ethnic groups.

**Results:**

The FLAME model, incorporating clinical and contextual-level SDoH, achieved an AUROC of 0.66. The clinical-only model performed slightly better (AUROC of 0.67), while the SDoH-only model had lower performance (AUROC of 0.54). SHAP analysis identified headache, organic brain syndrome, and pyuria as key predictors. Causal learning revealed interactions between clinical factors and contextual-level SDoH. Fairness assessments showed no significant biases across groups.

**Discussion:**

FLAME offers a fair and interpretable approach to predicting SLE flares, providing meaningful insights that may guide future clinical interventions.

**Conclusions:**

FLAME shows promise as an EHR-based tool to support personalized, equitable, and holistic SLE care.

## Introduction

Systemic lupus erythematosus (SLE) is a complex, chronic autoimmune disease with diverse clinical symptoms affecting multiple organ systems, with women being 9 times more likely than men to be affected.^1^^,^^2^ In the United States, SLE affects approximately 204 000 individuals,^3^ with significant differences in prevalence observed across racial and ethnic groups. For example, Non-Hispanic Blacks (NHB) are 4 times more likely to develop SLE compared to Non-Hispanic Whites (NHW),^4^ while Hispanics individuals have doubled the risk compared to their NHW counterparts.^5^ Moreover, non-White individuals with SLE tend to have more severe disease and worse outcomes, leading to significant disparities.^6^^,^^7^ These disparities underscore the importance of examining both clinical and contextual factors, including social determinants of health (SDoH), in understanding and managing SLE. Contextual-level SDoH refers to factors measured from an individual’s surroundings, including both social and physical environments, such as neighborhood characteristics, healthcare quality, and the built environment.^8^

Flares, or periods of increased SLE activity with worsening of symptoms, occur in 65%-70% of patients with S LE in any given year.^9^ Accurate flare recognition and prediction are crucial for evaluating responses to treatment and clinical outcomes, and guiding therapy. Recent studies identified several key predictive factors for flares of SLE. Petri et al^9^ identified elevated anti-dsDNA antibody levels, low complement levels (C3/C4), high erythrocyte sedimentation rate, anemia, lymphopenia, and high levels of interferon-regulated chemokines as significant predictors. Ugarte-Gil et al^10^ found that high post-baseline SLE Disease Activity Index scores (SLEDAI, calculated with clinical factors) and use of azathioprine were significant predictors. Inês et al^11^ identified younger age at diagnosis, prior lupus nephritis, and baseline immunosuppressive treatment as predictors of flares. Richardson^12^ explored environmental triggers such as air quality, employment status, and income that may cause epigenetic changes leading to flares. Goetz et al^13^ identified healthcare utilization factors, such as inpatient admissions and outpatient visits, as predictors.

Despite recognizing these predictors of disease flare, integrating predictive modeling into clinical practice remains challenging due to the logistics of adding to the information overload and administrative burden physicians face,^14^ shortages of healthcare workers,^15^^,^^16^ and limited patient engagement.^15^^,^^16^ Research on flares of SLE and disease management has seldom incorporated a holistic view that includes both clinical and SDoH factors. This oversight is particularly critical for SLE, given its complex and life- and organ-threating nature, substantial variability, and clear impact of SDoH factors.^7^^,^^17^^,^^18^ A more personalized and automated approach to both prediction and intervention for treating patients with SLE is needed.

The advent of real-world data (RWD) sources, such as electronic health records (EHRs) and administrative claims, coupled with advancements in artificial intelligence (AI), particularly machine learning (ML) techniques, for analyzing RWD, offer new opportunities for developing personalized patient care tools. These tools can help physicians improve health outcomes and equity by predicting disease activities and suggesting actionable interventions.^19^^,^^20^ However, many ML-based studies in healthcare have not adequately addressed biases inherent in observational RWD. These biases include the underrepresentation of certain patient groups, such as racial-ethnic minority groups, women, the elderly, and those from socioeconomically disadvantaged backgrounds. Not addressing these biases risks perpetuates existing disparities and inequities.^21^ Additionally, the opaque nature of ML algorithms often hinders their application and acceptability in clinical settings.^22^ Explainable AI techniques and causal structure learning approaches, such as the Peter-Clark (PC) algorithm,^23^ are essential for understanding the complex interplay between various predictive factors, thereby enhancing the fairness and transparency of ML models.

## Objective

In this study, we introduce FLAME (**FLA**re **M**achine learning prediction of SL**E**), an innovative ML pipeline leverage RWD from the University of Florida Health (UF Health) EHRs to accurately identify variables impacting flares in patients with SLE with stable disease and to fairly detect those at higher risk. By integrating both clinical characteristics and contextual-level SDoH, and prioritizing model fairness and explainability, we aimed to identify key causal factors that can be targeted for interventions. Our long-term goal is to develop an EHR-based, individualized SLE management platform that integrates both clinical and social risk factors to enhance patient care.

## Materials and methods

### Study design and population

**Figure 1.**
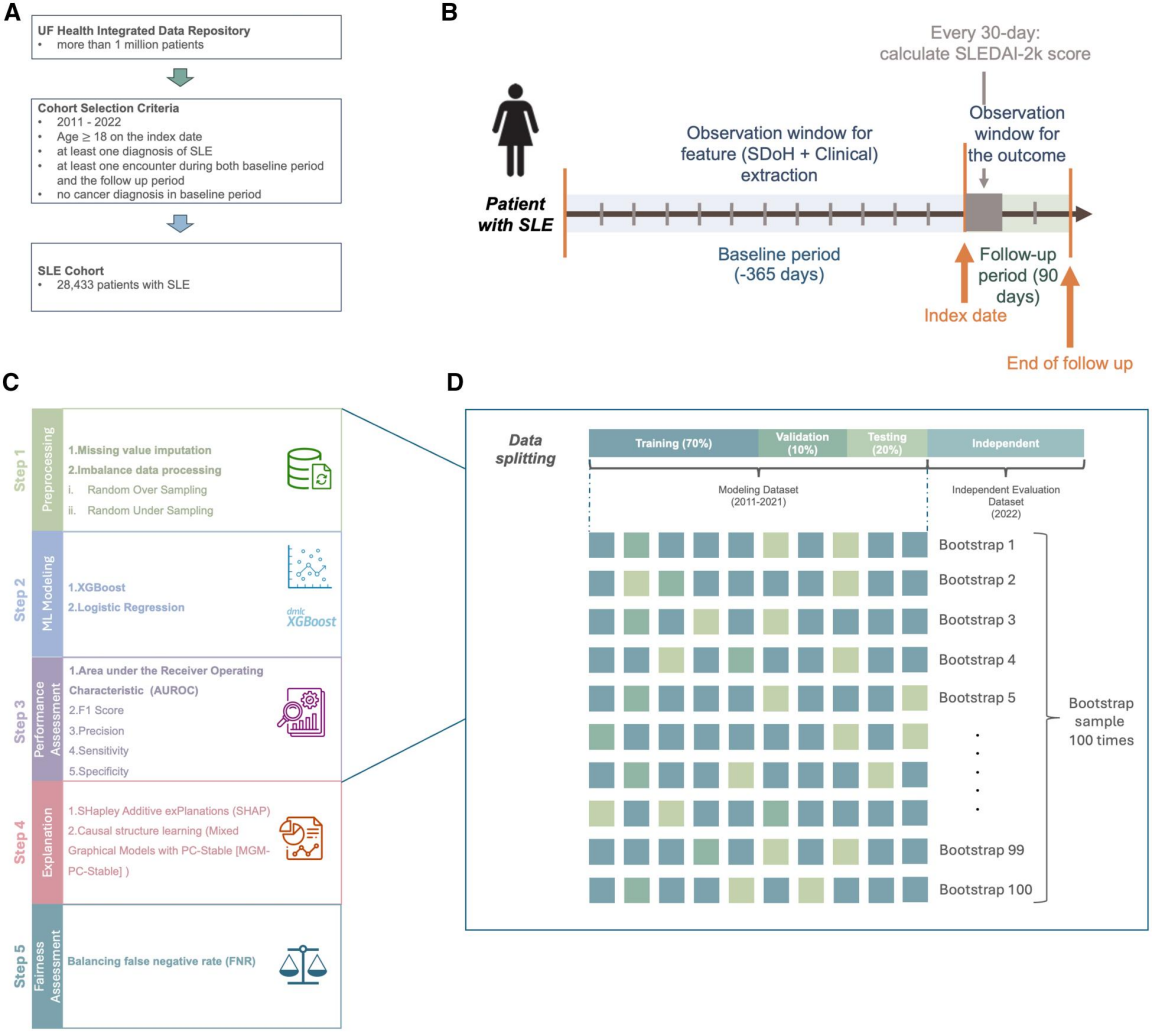
The overall workflow behind the FLAME pipeline. (A) Shows the cohort selection process. (B) Illustrates the patient timeline. (C) Outlines the FLAME pipeline, including 5 primary steps: preprocessing, machine learning modeling, performance assessment, explanation, and fairness assessment. (D) Displays the data splitting strategy, showing a modeling set (2011-2021) and an independent evaluation set (2022). The modeling set was further divided into training (70%), validation (10%), and testing (20%) sets, with 100 bootstrapped samples used for model robustness.

This study was approved by University of Florida Institutional Review Board (IRB202300125). We conducted a retrospective cohort study using the UF Health Integrated Data Repository EHRs from 2011 to 2022, linking the cohort with 675 contextual-level SDoH (eg, neighborhood unemployment rate and median income). The cohort included patients with SLE who (1) were aged 18 or older, (2) had a diagnosis of SLE confirmed by at least one International Classification of Diseases (ICD) code^24–26^ (ICD-9: 7100 and ICD-10: M32 or M320, M321, M3210—M3215, M3219, M328, M32.9), validated with a sensitivity of 100%, (3) had at least one encounter during both baseline and follow-up periods, (4) had a minimum of 3 months of follow-up after the index date, and (5) had no cancer diagnosis. Given the likelihood of multiple physician visits every year for patients with SLE, we defined the index date as a randomly selected outpatient encounter between the first recorded diagnosis of SLE and the second-last encounter in UF Health to mimic the real-world application of our FLAME prediction tool. We traced back 1 year before the index date as the baseline period to collect predictor information and followed up for 3 months to collect outcome (ie, flare occurrence) information (Figure 1A). The 3-month window was selected based on clinical relevance and preliminary model development considerations. Clinically, this interval aligns with typical follow-up schedules for patients with SLE, who are often reviewed every 1-3 months.^27^^,^^28^ This timeframe supports timely intervention and practical model application.

### Study outcome

The study outcome was the 3-month occurrence of flares, defined as episodes of increased SLE activity, characterized by symptoms worsening. Flare occurrence was determined using the SLEDAI 2000 (SLEDAI-2K) score, a validated EHR-based indicator of SLE disease activity by Goetz et al.^13^^,^^29^ To distinguish true disease worsening from ongoing baseline activity, we calculated the SLEDAI-2K score for each 30-day period during both baseline (ie, 12 months pre-index) and follow-up periods. Each patient’s baseline disease activity was defined as their median SLEDAI-2K score over the baseline period, ensuring that persistent but stable disease activity did not contribute to flare misclassification.

A flare in a given month was defined as an increase of 4 or more points above this baseline (Figure 1B),^30^ a threshold that has been validated in prior studies to reflect clinically meaningful worsening. Flare occurrence within 3 months post-index was recorded as a binary variable, indicating the presence or absence of at least one flare during that period. Each patient was then assigned a predicted risk score, representing the probability of experiencing at least one flare within 3 months of the index date, using the FLAME model.^31^ We then ranked and divided all patients’ risk scores into 11 groups (top first to fifth percentile, top sixth to tenth percentile, and subsequent deciles) to examine actual flare occurrence rates within 3 months by risk group. This stratification was used to help identify patients at the highest risk of flare and to illustrate how risk was distributed across the population, which may inform future efforts to define a clinically meaningful threshold for implementation.

### Covariates

We collected a comprehensive set of demographic, clinical, and contextual-level SDoH variables at baseline to assess their impact on the occurrence of flares in patients with SLE (see Supplementary Material S1 for details). All clinical variables were identified in EHRs using ICD codes and were selected based on clinical expertise and existing literature.^13^^,^^32^ All predictor variables were extracted exclusively from the 12-month pre-index baseline period to ensure that only pre-flare risk factors were included. No variables recorded during the follow-up period were used as predictors. We aimed to identify key causal factors that can be targeted for interventions, ensuring that the selected predictors contribute meaningfully to risk stratification and treatment decision-making.

### Development of ML pipeline for FLAME

The main goal of this analytics pipeline is to predict flare in patients with SLE with stable disease using both contextual-level SDoH and clinical risk factors in the SLE patient cohort built upon UF Health EHRs. Figure 1C and D show our overall analytics pipeline. First, we imputed missing data and then adopted balance processing techniques (Step 1. Preprocessing). Then we trained 2 ML models, namely XGBoost and logistic regression, using grid search cross-validation to optimize hyperparameters (Step 2. ML Modeling). XGBoost was selected because of its effectiveness in decision tree-based modeling, robustness to multicollinearity, and consistent predictive performance across diverse research domains. Logistic regression was included as a widely used and interpretable linear model for estimating event probabilities. To address multicollinearity and perform feature selection, we applied regularization techniques, including Least Absolute Shrinkage and Selection Operator (LASSO), Ridge, and elastic net penalties. These models were selected to balance predictive performance and interpretability, which is particularly important in clinical applications using structured EHR data. After that, we evaluated the model prediction performance using several metrics (eg, the Area Under the Receiver Operating Characteristic [AUROC]) on the independent testing dataset (Step 3. Performance Assessment) and utilized explainable AI and causal structure learning techniques, including SHapley Additive exPlanations (SHAP)^33^ and the Mixed Graphical Models with PC-Stable (MGM-PC-Stable),^34–37^ to identify important causal factors contributing to the outcome. We then examined the temporal distribution of the top 10 clinical features across different baseline and follow-up periods, visualizing how these features evolved using a Sankey circular diagram^38^ (Step 4. Explanation). Finally, in Step 5, we assessed the algorithmic fairness using the equality of opportunity metric, measured by the false-negative rate (FNR) to evaluate fairness across racial and ethnic groups. For detailed information on each step, see Supplementary Material S2.

## Results

### Descriptive statistics of the study cohort

Our final analysis comprised 28 433 eligible patients with SLE. Table 1 highlights the study cohort’s demographics and clinical characteristics.

**Table 1.** Summary of demographic and key clinical variables of the study cohort.

Name	Overall (n = 28 433)	NHW (n = 11 117, 39.1%)	NHB (n = 7475, 26.29%)	Hispanic (n = 7495, 26.36%)	Others (n = 1172, 4.12%)	Unknown (n = 1174, 4.13%)	*P*-value
**Age_at_diagnosis (SD** ^a^ **)**	46.31 (15.26)	48.50 (15.44)	43.81 (14.56)	44.99 (14.97)	44.57 (15.76)	51.74 (14.94)	<.001*
**Sex**							.074
Female	25412 (89.38%)	9892 (88.98%)	6672 (89.26%)	6803 (90.77%)	1037 (88.48%)	1008 (85.86%)	
**Preferred language**							.639
English	26078 (91.72%)	10907 (98.11%)	7363 (98.50%)	5564 (74.24%)	1103 (94.11%)	1141 (97.19%)	
Spanish	1845 (6.49%)	81 (0.73%)	11 (0.15%)	1719 (22.94%)	27 (2.30%)	7 (0.60%)	
Others	510 (1.79%)	129 (1.16%)	101 (1.35%)	212 (2.83%)	42 (3.58%)	26 (2.21%)	
**Alopecia**	156 (0.55%)	46 (0.41%)	53 (0.71%)	41 (0.55%)	12 (1.02%)	4 (0.34%)	.070
**Increased DNA binding** ^b^	312 (1.10%)	113 (1.02%)	106 (1.42%)	70 (0.93%)	20 (1.71%)	3 (0.26%)	.380
**Arthritis**	2093 (7.36%)	838 (7.54%)	560 (7.49%)	505 (6.74%)	77 (6.57%)	113 (9.63%)	<.001*
**Cranial nerve disorder**	214 (0.75%)	84 (0.76%)	41 (0.55%)	66 (0.88%)	14 (1.19%)	9 (0.77%)	<.001*
**Cerebral vascular accident**	1357 (4.77%)	542 (4.88%)	417 (5.58%)	298 (3.98%)	34 (2.90%)	66 (5.62%)	<.001*
**Fever**	1822 (6.41%)	614 (5.52%)	562 (7.52%)	498 (6.64%)	68 (5.80%)	80 (6.81%)	<.001*
**Headache**	5629 (19.80%)	2300 (20.69%)	1416 (18.94%)	1515 (20.21%)	192 (16.38%)	206 (17.55%)	<.001*
**Hematuria**	1767 (6.21%)	653 (5.87%)	478 (6.39%)	523 (6.98%)	60 (5.12%)	53 (4.51%)	<.001*
**Leukopenia**	2322 (8.17%)	697 (6.27%)	889 (11.89%)	608 (8.11%)	123 (10.49%)	5 (0.43%)	.022*
**Low complement** ^c^	202 (0.71%)	48 (0.43%)	79 (1.06%)	65 (0.87%)	10 (0.85%)	0 (0.00%)	.184
**Mucosal ulcers**	783 (2.75%)	346 (3.11%)	194 (2.60%)	182 (2.43%)	34 (2.90%)	27 (2.30%)	<.001*
**Myositits**	934 (3.28%)	412 (3.71%)	271 (3.63%)	172 (2.29%)	35 (2.99%)	44 (3.75%)	<.001*
**Organic brain syndrome**	3900 (13.72%)	1757 (15.80%)	1056 (14.13%)	760 (10.14%)	125 (10.67%)	202 (17.21%)	<.001*
**Pericarditis**	507 (1.78%)	125 (1.12%)	204 (2.73%)	147 (1.96%)	18 (1.54%)	13 (1.11%)	<.001*
**Pleurisy**	223 (0.78%)	82 (0.74%)	73 (0.98%)	53 (0.71%)	2 (0.17%)	13 (1.11%)	.003*
**Proteinuria**	1579 (5.55%)	396 (3.56%)	602 (8.05%)	446 (5.95%)	73 (6.23%)	62 (5.28%)	<.001*
**Psychosis**	2807 (9.87%)	1251 (11.25%)	815 (10.90%)	527(7.03%)	93 (7.94%)	121 (10.31%)	<.001*
**Pyuria**	3893 (13.69%)	1477 (13.29%)	980 (13.11%)	1119 (14.93%)	148 (12.63%)	169 (14.40%)	<.001*
**Rash**	4968 (17.47%)	1710 (15.38%)	1581 (21.15%)	1238 (16.52%)	219 (18.69%)	220 (18.74%)	<.001*
**Urinary casts**	570 (2.00%)	219 (1.97%)	171 (2.29%)	143 (1.91%)	17 (1.45%)	20 (1.70%)	<.001*
**Seizure**	1800 (6.33%)	798 (7.18%)	527 (7.05%)	347 (4.63%)	52 (4.44%)	76 (6.47%)	<.001*
**Thrombocytopenia**	1628 (5.73%)	544 (4.89%)	565 (7.56%)	426 (5.68%)	77 (6.57%)	16 (1.36%)	<.001*
**Vasculitis**	531 (1.87%)	187 (1.68%)	167 (2.23%)	137 (1.83%)	26 (2.22%)	14 (1.19%)	<.001*
**Visual disturbance**	1283 (4.51%)	500 (4.50%)	341 (4.56%)	346 (4.62%)	51 (4.35%)	45 (3.83%)	<.001*
**Baseline SLEDAI-2K score** ^d^ **mean (SD**^a^**)**	1.14 (2.77)	1.20 (2.89)	1.16 (2.79)	1.06 (2.65)	1.19 (2.73)	0.93 (2.40)	<.001*
**3-month flare prevalence**	5238 (18.42%)	2053 (18.47%)	1432 (19.16%)	1337 (17.84%)	181 (15.44%)	235 (20.02%)	.010*

a Standard deviation.

b Increased DNA binding by Farr assay above normal range for testing laboratory.

c Decrease in CH50, C3, or C4 below the lower limit of normal for testing laboratory.

d SLE Disease Activity Index 2000 scores.

* Statistical significance.

The overall mean age at diagnosis was 46.3 years, with a predominantly female cohort (89.38%), consistent with the known sex distribution of SLE. A significant majority of the cohort (91.72%) preferred English, with 6.49% speaking Spanish, and 1.79% speaking other languages. The racial/ethnic distribution was as follows: 39.10% NHW, 26.29% NHB, 26.36% Hispanic, 4.12% other races and ethnicities, and 4.13% unknown. NHB and Hispanic patients were generally younger than NHW patients (43.81 and 44.00 years vs 48.50 years, respectively).

At baseline, the overall mean SLEDAI-2K score was 1.14 with a standard deviation of 2.77, showing variation across racial and ethnic groups with scores ranging from 0.93 to 1.20. During the 3-month follow-up period, 5238 patients (18.42%) experienced at least one flare. The observed 3-month flare prevalence also varied by racial and ethnic groups, from 15.44% among patients categorized as “Other” to 20.02% among those with unknown race or ethnicity.

We also examined 24 clinical variables relevant to SLE with widely varying prevalences: Headache (Affected 19.8% overall), Rash (17.47%), Pyuria (13.69%), Organic Brain Syndrome (13.72%), Psychosis (9.87%), Leukopenia (8.17%), Arthritis (7.36%), Fever (6.41%), Seizures (6.33%), Hematuria (6.21%), Thrombocytopenia (5.73%), Proteinuria (5.55%), Cerebrovascular accident (CVA) (4.77%), Visual Disturbance (4.51%), Myositis (3.28%), Mucosal Ulcers (2.75%), Urinary Casts (2.00%), Vasculitis (1.87%), Pericarditis (1.78%), Increased DNA level (1.10%), Pleurisy (0.78%), Cranial Nerve Disorder (0.75%), and Low complement (0.71%).

### FLAME flare prediction models in patients with SLE

**Figure 2.**
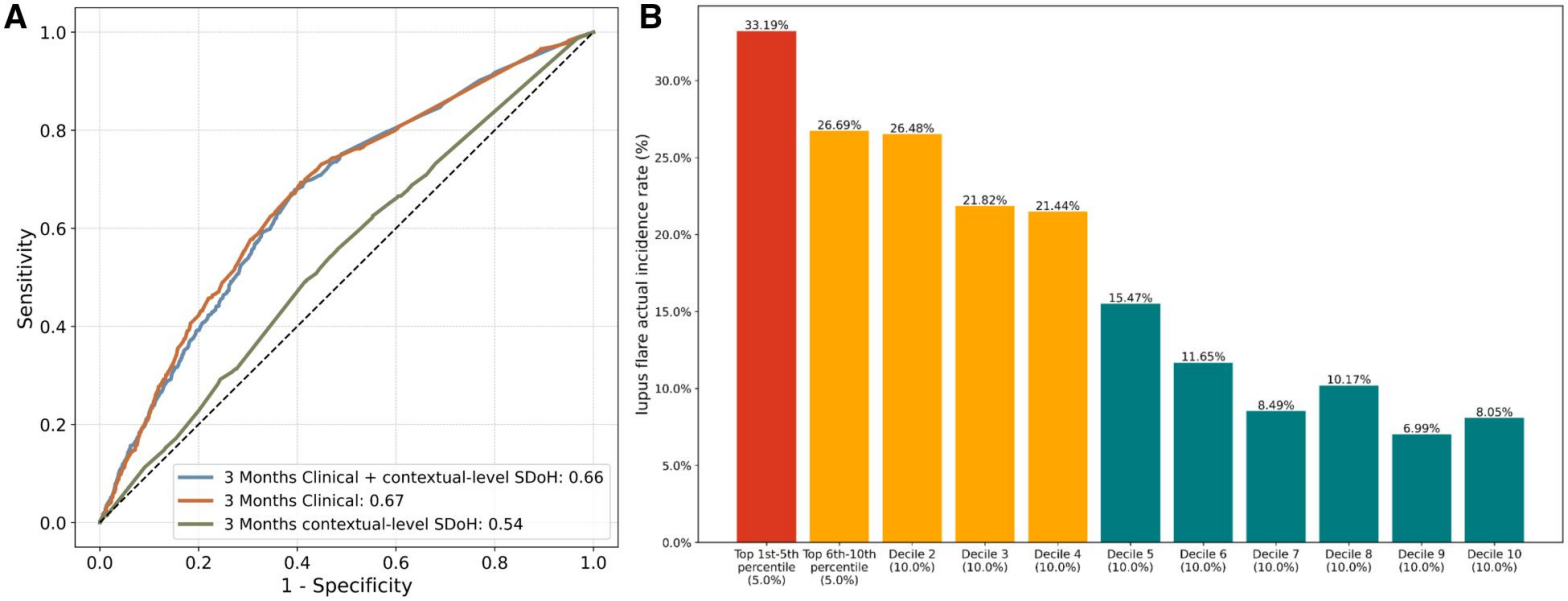
Three-month prediction of SLE flares using XGBoost with random oversampling. (A) Shows the 3-month area under the curve (AUROC) of XGBoost with random oversampling. The performance of the model was evaluated with different sets of features (clinical-only, contextual-level SDoH-only, and clinical factor and contextual-level SDoH) for predicting flares in patients with SLE over a 3-month period. (B) Shows the figure of 3-month flare risk by FLAME decile with XGBoost with oversampling. This figure presents the actual incidence rate of lupus flares over a 3-month period, stratified by deciles from the FLAME model. Each decile represents a risk group predicted by the XGBoost model with random oversampling.

We evaluated both XGBoost and logistic regression algorithms. Figure 2A presents the ROC curves illustrating the performance of the XGBoost-based predictive model with random oversampling, evaluated with different feature sets to predict 3-month flares in patients with SLE. In the XGBoost model, using both clinical and SDoH variables achieved an AUC of 0.6638. The clinical-only model yielded a slightly higher AUC of 0.67, while the contextual-level SDoH-only model had a significantly lower AUC of 0.54. The results from the logistic regression algorithm showed quite similar results (Figure S1).

In the independent evaluation dataset, we stratified the predicted 3-month flare risk by decile using the XGBoost-generated FLAME pipeline, demonstrating excellent utility in identifying individuals at risk of flare. For example, the 3-month risk of flare in the top 5% of the patient cohort, based on the XGBoost model with oversampling, was 33.19%, more than 4 times that of the bottom decile. The risk in the top 10% of the cohort added up to 59.88% (Figure 2B). Similarly, in the logistic regression model (Figure S2), the top 5% of predicted cases accounted for 28.94% of the 3-month flare risk, and the top 10% accounted for 59.87% of the risks.

### Explainable AI to identify important features contributing to FLAME predicting flares in patients with SLE

**Figure 3.**
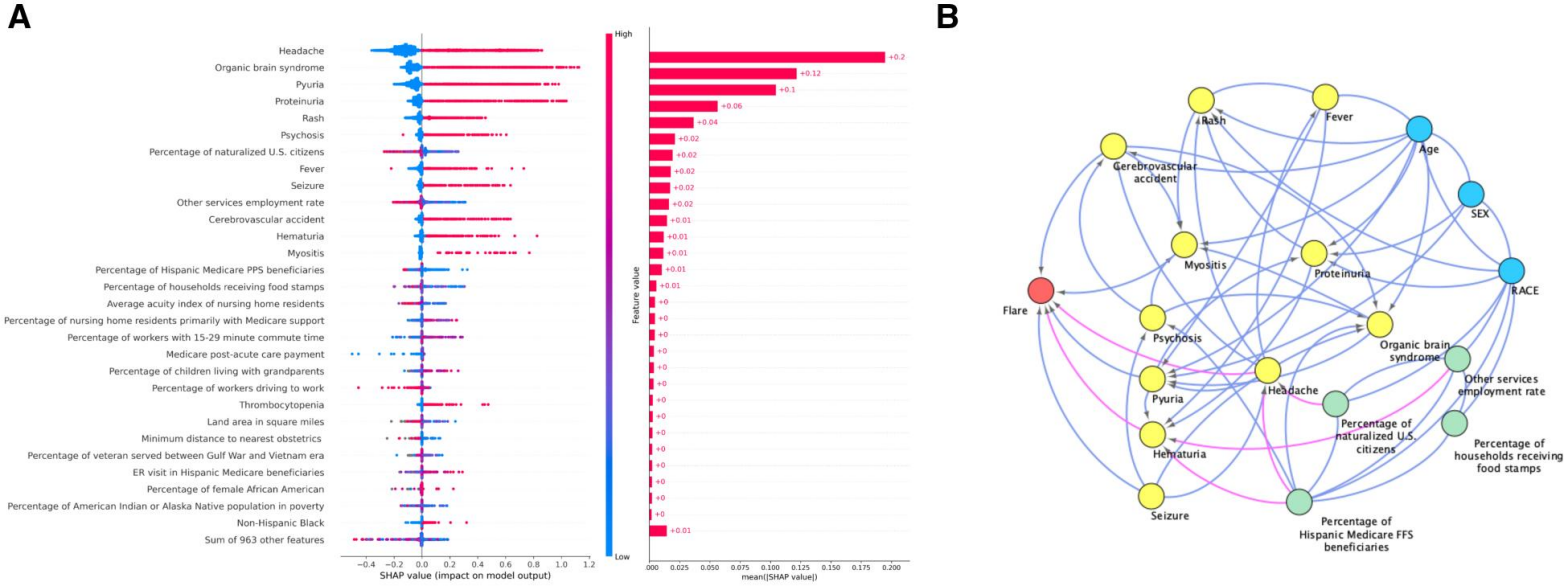
Feature importance and network analysis for predicting SLE flares with XGBoost model. (A) Displays feature importance analysis with SHAP values on the XGBoost model with random oversampling. SHAP values are from the original XGBoost. We removed the features with an “unknown” category. (B) Shows a causal graph generated by MGM-PC-Stable on the independent evaluation dataset. The blue nodes present demographics, the green nodes stand for contextual-level SdoH, the yellow nodes represent the clinical variables, and the red node indicates the outcome. The pink edges represent the indirect relationships between SDoH and outcome.

Based on the analysis presented in Figure 3A, the XGBoost results indicate that “headache” is the most significant predictor of flares in patients with SLE within a 3-month period, followed by “organic brain syndrome” and “pyuria.” This finding is consistent across both the XGBoost and Logistic Regression models (Figure S3), which were trained on a full dataset with Random Over Sampling to address class imbalance. Other important features included “proteinuria,” “rash,” “psychosis,” and a contextual-level variable: “percentage of the population consisting of US citizens by naturalization,” which was identified as important in the model’s prediction of flares.

Figure 3B displays our exploratory analysis with causal structure learning, applying the MGM-PC-Stable method to build the causal DAGs of the key features. In this analysis, we identified 3 demographic variables, 11 clinical variables, and 4 contextual-level SDoH, resulting in a causal graph with 19 nodes (including the outcome of flare) and 58 edges representing direct or indirect relationships between these variables that emerged in the causal network. Among the clinical variables, 6—headache, pyuria, hematuria, seizure, cerebrovascular accident, and myositis—were found to be causally related to flares in patients with SLE, either directly or indirectly. Additionally, pyuria was influenced by fever, age at diagnosis, sex, organic brain syndrome, and headache. This finding aligns with the insights derived from SHAP values obtained from both XGBoost and logistic regression models. These models suggest that contextual-level SDoH, specifically the percentage of naturalized US citizens and the higher percentage of Hispanic Medicare Prospective Payment System beneficiaries leads to a higher tendency of headache, contributing to flares of SLE.

**Figure 4.**
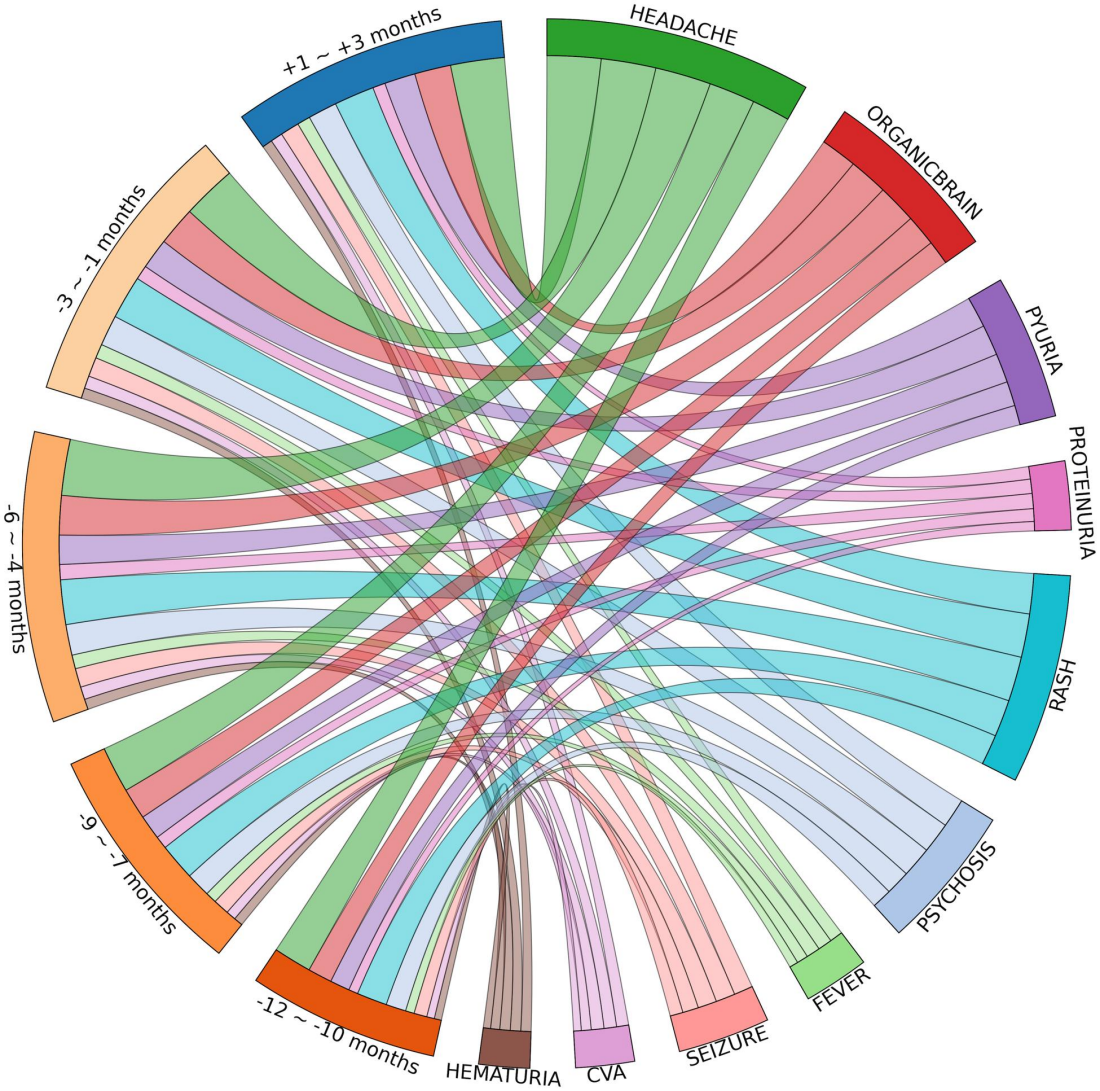
Distribution of top 10 clinical variables in XGBoost ROS SHAP analysis for 3-month baseline, 6-month baseline, 9-month baseline, 12-month baseline, and 3-month follow-up data. This figure presents a Sankey circular diagram illustrating the longitudinal evolution of the top 10 clinical variables of SLE flares across multiple time windows. The left side of the diagram represents earlier time periods (-12 to -10 months, -9 to -7 months, -6 to -4 months, and -3 to -1 months before flare onset), while the right side represents the post-flare period (+1 to +3 months). The width of each flow represents the relative frequency of a given clinical feature at each time window. Key predictors such as headache, organic brain syndrome, pyuria, proteinuria, and rash exhibit varying prevalence patterns over time.

To examine the distribution of key clinical predictors associated with SLE flares, we visualized the longitudinal evolution of the top 10 features, including “headache,” “organic brain,” “pyuria,” “proteinuria,” “rash,” “psychosis,” “fever,” “seizure,” “CVA,” and “hematuria,” using a Sankey diagram (Figure 4). We also summarized the numerical distribution of these variables across 12-month, 9-month, 6-month, and 3-month pre-flare periods, as well as the 3-month post-flare period in Table S3. “Headache” and “organic brain” syndrome showed a steady increase leading up to the flare, peaking in the -6 to -4 month and -3 to -1 month periods, and remained elevated post-flare (+1 to +3 months). Similarly, “pyuria,” “rash,” and “psychosis” increased consistently before flare onset, though pyuria remained stable post-flare rather than decreasing. In contrast, “proteinuria,” “fever,” and “seizure” exhibited moderate increases before the flare and remained relatively stable afterward. Finally, “CVA” and “hematuria” demonstrated slower increases pre-flare and remained stable in the post-flare period.

### Fairness assessment and mitigation

We assessed fairness in the FLAME model by evaluating equality of opportunity across racial and ethnic groups, specifically NHW, NHB, and Hispanic populations, using FNR balance as the primary metric. The FNR represents the probability of incorrectly predicting no flare in an individual who actually experiences a flare. Figures S4 and S5 show the FNR curves for these groups in the XGBoost and logistic regression models, respectively, with nearly indistinguishable results, indicating similar predictive performance across these groups. Additionally, FNR balance calculations (using NHW as the reference group) across various feature sets (Table 2) uniformly fell within the range of statistical fairness (0.8-1.25). This finding suggests that the FLAME model does not exhibit substantial disparities in FNR for either NHB or Hispanic individuals compared to NHW.

**Table 2.** Opportunity of equality measured by false-negative rate by different models on various feature sets.

Black and White	Clinical + SDoH 3 months	Clinical 3 months	SDoH 3 months
XGBoost	0.91	0.90	1.03
Logistic regression	0.89	0.90	0.80

**Hispanic and White**	**Clinical + SDoH 3 months**	**Clinical 3 months**	**SDoH 3 months**

XGBoost	1.04	1.03	1.24
Logistic regression	1.00	1.02	0.99

SDoH: social determinants of health.

## Discussion

In this study, we developed a fair and explainable ML pipeline, FLAME, to predict patients with SLE at high risk of flares. Using UF Health EHR data from 28 433 patients with SLE, we incorporated both clinical variables and contextual-level SDoH. The FLAME model, integrating both data sources, achieved an AUROC of 0.66. The clinical-only model performed slightly better (AUROC of 0.67), while the contextual-level SDoH-only model showed lower performance (AUROC of 0.54). SHAP analysis identified headache, organic brain syndrome, and pyuria as key predictors. Causal learning revealed interactions between clinical factors and contextual-level SDoH. Fairness assessments indicated no significant disparities in model performance across racial and ethnic groups. These findings demonstrate that FLAME is a promising tool for accurately and fairly identifying patients with SLE at higher risk for flares, offering explainable insights for future interventions.

Our model identified several symptom-level features, such as headache, organic brain syndrome, and pyuria, which may serve as early warning signals of flare risk and deserve further investigation due to their accessibility in routine care. Prior studies predicting SLE flares remain limited. Petri et al^9^ conducted a post hoc analysis of 562 patients from phase III belimumab trials and found that high baseline disease activity, anti-dsDNA positivity, proteinuria, elevated BLyS levels, and BILAG organ involvement were independent predictors of moderate-to-severe flares. Inês et al^11^ prospective cohort study of 202 SLE patients and identified younger age at diagnosis (≤25 years), prior lupus nephritis, and immunosuppressive treatment history as independent predictors. Goetz et al^13^ developed a claims-based algorithm using data from 2427 patients and identified inpatient admission, outpatient visits, MRI use, ER visits, and rheumatology visit frequency as key predictors. Notably, it is the only study that identified a similar symptom-related predictor, headache, although the association was not statistically robust.

While our analysis revealed that contextual-level SDoH did not substantially improve the predictive performance of the FLAME model for flares of SLE, their importance should not be overlooked. The causal structure learning analysis uncovered key roles for SDoH variables in several causal pathways leading to flares of SLE. For instance, neighborhood-level factors such as the percentage of naturalized US citizens and the proportion of Hispanic Medicare Fee-for-Service beneficiaries were found to influence the prevalence of headaches, which in turn correlates to flares of SLE. This finding underscores the complex interplay between social factors and clinical manifestations in SLE.

Despite their limited direct predictive power in this model, the inclusion of contextual-level SDoH in ML models like FLAME is crucial for several reasons. First, it provides a more holistic understanding of the factors influencing flares of SLE, capturing the broader socioeconomic and environmental context in which patients live. Second, identifying these causal pathways offers potential targets for intervention that extend beyond traditional clinical approaches. Public health initiatives or community-based programs addressing these contextual factors could complement medical treatments in managing SLE. Lastly, the incorporation of contextual-SDoH in predictive models aligns with the growing recognition of health equity as a central goal in healthcare. By considering these factors, we can develop more comprehensive and equitable strategies for SLE management, potentially reducing disparities in health outcomes among different patient populations.

Our fairness assessment of the FLAME model demonstrated robust (ie, consistent and reliable) fairness across race and ethnicity groups, which is crucial for ensuring equitable healthcare interventions. The main models, incorporating both clinical and contextual-level SDoH factors, as well as the clinical-only models, exhibited fairness metrics within the commonly accepted range of statistical fairness (0.80 to 1.25)^39^ for race and ethnicity groups. This indicates that our primary predictive tools do not disproportionately disadvantage any particular group in terms of flare risk assessment. The model using only contextual-level SDoH showed a slightly higher FNR for Hispanic patients compared to NHW patients, though still within the statistical fairness range, suggesting the critical role of SDoH underlying ML bias and fairness. Nevertheless, the contextual-level SDoH-only models had low predictive utility, which is not intended for clinical use. This suggests that while contextual-level factors capture broader environmental influences, they may not fully account for patient-specific social and economic stressors that directly impact health. Future research should incorporate both contextual and individual-level SDoH, in the prediction of SLE adverse outcomes.

The clinical implications of our FLAME pipeline are significant. The model demonstrated excellent utility in identifying patients with SLE at high flare risk using real-world EHR data. Notably, the top 10% of the patient cohort that predicted by the FLAME model accounted for 59.88% of the risk of flares in a 3-month period, after adjusting for patients’ demographic and clinical characteristics. We found that the increased flare risk in SLE is largely attributable to clinical factors including headache, pyuria, hematuria, seizure, CVA, and myositis. Notably, headache, seizure, and new onset CVA are descriptors of disease activity that carry some of the highest weighted scores (8 points each) in the SLEDAI-2K measure.

The FLAME model has the potential to support clinical decision-making by identifying SLE patients at high risk of flares. In practice, the model could be used to prioritize high-risk patients for proactive care, such as more frequent monitoring, early follow-up, or timely therapeutic adjustments. By integrating clinical factors and contextual-level SDoH, and demonstrating equitable performance across racial and ethnic groups, FLAME may help clinicians improve disease management while advancing health equity. However, several barriers remain, including the need for timely and continuously updated EHR data, seamless integration of predictive tools into clinical workflows, and the generation of interpretable outputs that foster clinician trust and usability. Future work should validate the model using external datasets, incorporate individual-level SDoH, and explore automated machine learning approaches to enhance performance and streamline development. Addressing these barriers will be essential for translating predictive models like FLAME into real-world tools that support personalized and equitable SLE care.

Our study has several strengths. First, we leveraged a large, diverse cohort of 28 433 patients with SLE, incorporating both clinical and contextual-level SDoH variables to enhance the predictive performance of our model. Second, we employed explainable AI techniques (SHAP values) and causal structure learning (MGM-PC-Stable) to provide interpretable insights into flare prediction, identifying key clinical and contextual-SDoH that may be targeted for interventions. Third, we visualized the temporal distribution of top clinical predictors using a Sankey circular diagram, which allowed us to examine whether the evolution of these features before, during, and after flare onset aligned with clinical experience. This visualization enhances interpretability and provides a novel way to explore dynamic changes in disease activity. Lastly, we assessed algorithmic fairness using the equality of opportunity metric to ensure that our model performs equitably across racial and ethnic groups, an essential consideration for real-world implementation.

Our study has several limitations. First, the cohort of 28 433 patients with SLE was identified primarily through diagnosis codes, which may lead to diagnostic inaccuracies in primary care settings,^40^ including potentially false-positive cases. In the future, we plan to utilize advanced Natural Language Processing (NLP) to analyze clinical notes and improve cohort accuracy. Second, while our cohort from OneFlorida+ EHR was diverse, with over 26% Black and 26% Hispanic patients across rural and urban populations, this may limit the generalizability of our findings to other regions of the United States and internationally. Future research should aim to enhance the generalizability of FLAME through federated learning and geographically diverse data sources.^41^ Another limitation is that while we incorporated contextual-level SDoH, we did not include individual-level SDoH, which captures patient-specific social and economic conditions such as education, income, employment, and health behaviors.^8^ While contextual-level SDoH reflects broader community characteristics, they may not fully capture personal socioeconomic stressors, such as financial hardship, food insecurity, and social support, that directly influence flare risk.^7^ Future research should leverage NLP to extract individual-level SDoH from clinical notes and evaluate their impact on flare prediction models. Furthermore, the selection and tuning of the FLAME followed standard ML practices, limiting the search space for models and hyperparameters. Future efforts will incorporate automated ML pipelines to enhance model accuracy and reliability while simultaneously minimizing the time and resources required to develop the next-generation model. Finally, since some predictor variables, such as proteinuria, psychosis, and thrombocytopenia, are also components of SLEDAI-2K, they contribute to both risk estimation and flare determination. While flares were defined based on a relative increase in SLEDAI-2K, rather than the presence of individual components alone, there remains a possibility that the model captures ongoing disease activity rather than true flare prediction. Future studies should investigate alternative flare definitions and evaluate the impact of excluding overlapping predictors on model performance.

## Conclusion

Overall, this study presents FLAME, an ML pipeline for predicting flares of SLE by integrating clinical factors and contextual-level SDoH. Our findings demonstrate that FLAME achieves good predictive performance while maintaining fairness across racial and ethnic groups, addressing a critical need in the management of SLE. The model’s explainability, via SHAP analysis and causal structure learning, sheds light on the complex interplay between clinical and social factors in flares of SLE. While clinical variables were the strongest predictors, incorporating contextual-level SDoH revealed important causal pathways that could inform broader intervention strategies. Despite some limitations, FLAME represents a significant step towards more personalized, equitable, and holistic SLE care.

## Supplementary Material

ooaf072_Supplementary_Data

## Data Availability

The data underlying this article cannot be shared publicly due to privacy restrictions. The derived data generated in this research will be shared on reasonable request to the corresponding author.
