# Supplementary material for: A fair machine learning model to predict flares of systemic lupus erythematosus
Source: JAMIA Open. 2025 Jul 26;8(4):ooaf072. doi: 10.1093/jamiaopen/ooaf072 (PMC12296391; doi:10.1093/jamiaopen/ooaf072)
Supplement: ooaf072_Supplementary_Data [file ooaf072_supplementary_data.docx]

**Supplemental materials**

1. S1: Covariates
2. S2: Detailed Steps of the ML Pipeline for FLAME Prediction
3. Table S1: Summary of contextual-level SDoH.
4. Table S2: Demographic and Clinical Characteristics of the Study Cohort Stratified by Flare Risk Deciles
5. Table S3: Performance metrics for XGboost and Logistic Regression
6. Table S4: Distribution of Top 10 clinical variables in XGBoost ROS SHAP analysis
7. Figure S1: Performance assessment by AUROC for the logistic regression model
8. Figure S2: Flare risk by FLAME decile using logistic regression on independent data
9. Figure S3: Feature importance analysis with SHAP values (Logistic regression model)
10. Figure S4: False negative rate (FNR) curve across different population groups using Xgboost
11. Figure S5: False negative rate (FNR) curve between different populations using logistic regression

**S1: Covariates**

**Demographics and clinical characteristics**

Demographic covariates included age, sex (e.g., female or male), race/ethnicity (e.g., NHW, NHB, and Hispanic), and preferred language. Clinical characteristics at baseline included common indicators of SLE activity, such as increased DNA binding, defined as levels exceeding the normal range for the testing laboratory as measured by the Farr assay, and arthritis, and arthritis. We also documented more specific conditions, including cranial nerve disorder, cerebrovascular accident (CVA), fever, and headache. Renal-related symptoms such as hematuria, proteinuria, and pyuria were also recorded, alongside hematological indicators like leukopenia and thrombocytopenia. Additional clinical manifestations that were noted included low complement levels, defined as decrease in CH50, C3, or C4 below the lower limit of normal for testing laboratory, as well as mucosal ulcers, myositis, organic brain syndrome, pericarditis, pleurisy, psychosis, rash, urinary cast, seizure, vasculitis, and visual disturbance. Patients’ residential zip codes were collected during the baseline period for linkage to contextual-level SDoH data.

**Contextual-level SDoH**

To capture the broader socio-economic and environmental factors that could influence health outcomes, we obtained 675 contextual-level SDoH measures related to the built and social environment, including information on social context (e.g., percentage of households receiving food stamps, household internet access status), economic context (e.g., household income and employment status of a given zip code are), education (e.g., percentage of people with income between 100% and 124% of the federal poverty level, percentage of veterans aged 18 to 64 living below the poverty line), physical infrastructure (e.g., air pollution measured by PM2.5, drought events), healthcare context (e.g., ambulances available at hospitals, self-harm mortality), and geography (e.g., rural-urban classification, population density of a county) from 24 well-validated sources (**Supplementary Table S1**) (e.g., American Community Survey [ACS], Area Health Resource Files [AHRF], amfAR Opioid & Health Indicators Database [AMFAR]). These measures, with varying temporal scales, were linked to each patient using their baseline residential address and 9-digit ZIP codes. We calculated area-weighted averages within a 250-mile buffer around each ZIP code's centroid, followed by time-weighted averages based on each individual’s residential history.

**S2: Detailed Steps of the ML Pipeline for FLAME Prediction**

**Step 1: Data preprocessing**

To build features for prediction models, we first imputed missing values using the mode value, and “unknown” label for the value equals to “UN”, for categorical variables and the mean for continuous variables. Next, we created dummy variables for the categorical variables.

**Step 2: Machine learning model development for FLAME**

We developed the FLAME model to predict SLE risk in SLE patients. Specifically, we aimed to predict the likelihood of flare occurrence within three months after the baseline using three sets of input features in addition to demographic features: (1) SDoH only, (2) clinical features only, and (3) a combination of SDoH and clinical features. We considered two widely used machine learning approaches: XGBoost and logistic regression. XGBoost, known for its effectiveness in decision-tree-based models, has consistently delivered accurate predictions across various research domains^1–6^. Logistic regression^7^, a robust and interpretable linear model, is commonly used for predicting event probabilities while accounting for influencing variables to provide clear and accurate results. Following machine learning best practices, we split the dataset into a modeling set (spanning data from 2011 to 2021) and an independent evaluation set (from 2022). The modeling set was further divided into training, validation, and testing sets in a 7:1:2 ratio. To optimize model parameters, we performed a five-fold cross-validation grid search on the training set and applied early stopping on the validation set to avoid overfitting.

**Step 3: Performance Assessment.**

We assessed the performance of each model using several metrics: area under the receiver operating characteristic curve (AUROC), F1 score, precision, recall, and specificity. To ensure robust performance estimates, we applied bootstrapping with 100 iterations on the modeling dataset, which included the training, validation, and testing sets. Standard deviation for each evaluation metric was calculated to account for potential variability in the results. The model with the highest AUROC was selected as the best-performance model. To address data imbalance before training, we employed random over-sampling (ROS), which increases the number of minority class samples.

**Step 4: Explainable AI**

First, we applied SHAP^8^ to identify significant SDoH and clinical features that were contributed to FLAME predicting flare occurrence in SLE patients. Following this, we employed the Mixed Graphical Models with PC-Stable (MGM-PC-Stable)^9–12^, a causal structure learning, to discern the potential causal relationships among these identified pivotal clinical and SDoH features in a directed acyclic graph (DAG) format. This approach helped us understand how these features collectively influence the likelihood of flare occurrence in SLE patients. We then examined the temporal distribution of the top ten clinical features across different baseline and follow-up periods. To visualize how these features evolved over time, we used a Sankey circular diagram^13^ to track feature frequency across five time windows: 12-10 months pre-index, 9-7 months pre-index, 6-4 months pre-index, 3-1 months pre-index, and 1-3 months post-index. This analysis enabled us to assess whether the temporal patterns of flare-associated features aligned with clinical expectations, providing insights into how risk factors behave before, during, and after flare onset.

**Step 5: Algorithmic fairness optimization**

To evaluate the fairness of the FLAME model, we utilized the equality of opportunity metric (measured by false negative rate [FNR]). This metric assesses the rate at which individuals are incorrectly classified as low risk when they are actually at high risk, focusing on different gender and racial-ethnic groups, particularly NHB and Hispanic versus NHW populations^14^. This focus is crucial because preventing misclassification in these groups is vital due to the adverse health outcomes associated with flare events. Our goal was to ensure that the FLAME model does not exhibit a higher FNR within these disadvantaged groups compared to NHW. Given the lack of a universally agreed-upon fairness threshold, we deemed the equality of opportunity within the range of 0.80 to 1.25 as statistically fair, flagging any values beyond this interval^15^.

**Table S1: Summary of contextual-level SDoH.**

| **Category** | **Data Source** | **Time period** | **Temporal scale** | **Number of variables** |
| --- | --- | --- | --- | --- |
| 1. Social context | ACS | 2009-2020 | cross-sectional | 104 |
|  | AHRF | 2009-2020 | 1 year | 3 |
| 2. Economic context | ACS | 2009-2020 | cross-sectional | 72 |
|  | AHRF | 2009-2020 | 1 year | 1 |
|  | SAIPE | 2018 | 10 years | 7 |
| 3. Education | ACS | 2009-2020 | cross-sectional | 10 |
| 4. Physical infrastructure | ACS | 2009-2020 | cross-sectional | 72 |
|  | AHRF | 2009-2020 | 1 year | 5 |
|  | NEPHTN | 2019-2020 | 3months | 15 |
|  | EPAA | 2019-2020 | 1 year | 14 |
|  | NOAAS | 2018-2019 | 1month | 14 |
|  | NOAAC | 2018-2019 | 1month | 48 |
|  | WUSTL | 2018-2019 | 1 year | 1 |
| 5. Healthcare context | ACS | 2009-2020 | cross-sectional | 51 |
|  | AHRF | 2009-2020 | 1 year | 30 |
|  | AMFAR | 2010-2011, 2013-2017. 2019-2020 | 1 year | 24 |
|  | CDCW | 2018-2020 | 1 year | 8 |
|  | CHR | 2009-2020 | cross-sectional | 3 |
|  | MP | 2009-2011, 2013-2020 | 1 year | 3 |
|  | NHC | 2013-2016. 2018-2020 | 3months | 5 |
|  | HHC | 2018 | 1 year | 6 |
|  | HIFLD | 2018 | 1 year | 6 |
|  | IHS | 2018-2019 | 1 year | 2 |
|  | LTC | 2018-2019 | 1 year | 15 |
|  | MGV | 2018-2019 | 1 year | 16 |
|  | MMD | 2018-2019 | 1 year | 47 |
|  | PC | 2018-2019 | 1month | 2 |
|  | POS | 2018-2019 | 1year | 71 |
| 6. Geography | AHRF | 2009-2020 | 1 year | 1 |
|  | CAF | 2009-2020 | 1 year | 14 |
|  | CEN | 2009-2016, 2018-2020 | 1 year | 3 |
|  | NCHS | 2009-2016, 2018-2020 | 1 year | 2 |

Abbreviations: ACS: American Community Survey; AHRF: Area Health Resource Files; AMFAR: amfAR Opioid & Health Indicators Database; CAF: County Adjacency File; CDCW: CDC WONDER (Wide-ranging Online Data for Epidemiologic Research); CEN: U.S. Census Bureau, TIGERweb and COVID-19 Demographic and Economic Resources; CHR: County Health Rankings; MP: Medicare Advantage State/County Penetration Files; NCHS: National Center for Health Statistics Urban-Rural Classification Scheme; NEPHTN: National Environmental Public Health Tracking Network; NHC: Nursing Home Compare; EPAA: Environmental Protection Agency; NOAAS: National Oceanic and Atmospheric Administration Storm Events Database; NOAAC: National Oceanic and Atmospheric Administration Climate; SAIPE: Census Bureau Small Area Income and Poverty Estimates; WUSTL: Washington University Regional Estimates of Chemical Composition of Fine Particulate Matter using a Combined Geoscience-Statistical Method with Information from Satellites, Models, and Monitors; HHC: Home Health Compare; HIFLD: Homeland Infrastructure Foundation-Level Data; IHS: Indian Health Service; LTC: Long-term Care: Facts on Care in the U.S. Public Use Data; MGV: Medicare Geographic Variation Public Use File; MMD: Mapping Medicare Disparities Tool; PC: Physician Compare; POS: Centers for Medicare and Medicaid Provider of Services File.

**Table S1** provides an overview of the contextual-level data sources, the time periods of data collection, and the temporal scales. All data were collected at the county spatial scale. The dataset includes 107 measures related to social context, primarily obtained from the American Community Survey (ACS) for the years 2009-2020, along with additional information from the Area Health Resource Files (AHRF). Economic context data consist of 80 measures, sourced from the ACS, AHRF and the Census Bureau's Small Area Income and Poverty Estimates (SAIPE), with time periods ranging from 2009 to 2020. Education-related variables include 10 measures from the ACS for 2009-2020. Physical infrastructure data include 169 measures collected from seven sources, such as the ACS, the Environmental Protection Agency (EPA), the National Environmental Public Health Tracking Network (NEPHTN), and the National Oceanic and Atmospheric Administration (NOAA), with temporal scales ranging from monthly to yearly with various time period. Healthcare context data consist of 289 variables, collected from 15 sources such as ACS, AHRF, amfAR Opioid & Health Indicators Database (AMFAR), and the Centers for Medicare and Medicaid Services (CMS) Provider of Services (POS) File, with varying time periods and scales. Finally, 20 geography-related measures were obtained from four sources such as the U.S. Census Bureau, the County Adjacency File (CAF), and the National Center for Health Statistics (NCHS), with data covering 2009 to 2020.

**Table S1** presents detailed information on the data sources, collection periods, and temporal scales for the contextual-level measures in this study, all collected at the county level spatial scale. The dataset includes 107 social context measures from the American Community Survey (ACS) for 2009-2020, along with Area Health Resource Files (AHRF) for . Economic context measures include 80 variables, also primarily from ACS, along with data from the Census Bureau's Small Area Income and Poverty Estimates (SAIPE). Education measures consist of 10 variables obtained from ACS for the same period. Physical infrastructure, with 169 variables, was drawn from sources such as ACS, the National Environmental Public Health Tracking Network (NEPHTN), and the Environmental Protection Agency (EPA), with data on factors such as air quality and natural events. Healthcare context includes 289 variables, sourced from ACS, the amfAR Opioid & Health Indicators Database, and CMS Provider of Services (POS) File, focusing on healthcare infrastructure and access. Lastly, the geography category, with 20 variables, was based on data from the County Adjacency File (CAF) and the National Center for Health Statistics (NCHS), capturing geographic features such as land area and population density. These measures, with varying temporal scales, provide a robust dataset to examine the effects of socio-economic, environmental, and healthcare factors on health outcomes.

**Table S2:** **Demographic and Clinical Characteristics of the Study Cohort Stratified by Flare Risk Deciles**

| **Name** | **Top 10 decile (n=471)** | **10-50 decile**  **(n=1887)** | **Bottom 50 decile**  **(n=2359)** | **p-value** |
| --- | --- | --- | --- | --- |
| Age_at_Diagnosis | 42.48 | 45.87 | 48.77 | <0.001* |
| **Sex** |  |  |  |  |
| Female | 431 (91.51%) | 1700 (90.09%) | 2107 (89.32%) | 0.135 |
| **Preferred language** |  |  |  |  |
| English | 448 (95.12%) | 1790 (94.86%) | 2189 (92.79%) | 0.319 |
| Spanish | 16 (3.40%) | 69 (3.66%) | 127 (5.38%) | 0.387 |
| Others | 7 (1.49%) | 28 (1.48%) | 43 (1.82%) | 0.752 |
| **Alopecia** | 6 (1.27%) | 13 (0.69%) | 15 (0.64%) | 1.000 |
| **Increased DNA binding** | 29 (6.16%) | 123 (6.52%) | 131 (5.55%) | 0.607 |
| **Arthritis** | 0 (0.00%) | 0 (0.00%) | 0 (0.00%) | 1.000 |
| **Cranial nerve disorder** | 8 (1.70%) | 27 (1.43%) | 12 (0.51%) | 0.006* |
| **Cerebral vascular accident** | 80 (16.99%) | 120 (6.36%) | 9 (0.38%) | <0.001* |
| **Fever** | 135 (28.66%) | 229 (12.14%) | 40 (1.70%) | <0.001* |
| **Headache** | 325 (69.00%) | 795 (42.13%) | 14 (0.59%) | <0.001* |
| **Hematuria** | 123 (26.11%) | 202 (10.70%) | 34 (1.44%) | <0.001* |
| **Leukopenia** | 93 (19.75%) | 221 (11.71%) | 208 (8.82%) | 0.013* |
| **Low complement** | 22 (4.67%) | 60 (3.18%) | 41 (1.74%) | 0.053 |
| **Mucosal ulcers** | 22 (4.67%) | 77 (4.08%) | 43 (1.82%) | 0.691 |
| **Myositits** | 12 (2.55%) | 26 (1.38%) | 1 (0.04%) | 0.587 |
| **Organic brain syndrome** | 287 (60.93%) | 337 (17.86%) | 2 (0.08%) | <0.001* |
| **Pericarditis** | 15 (3.18%) | 36 (1.91%) | 27 (1.14%) | 0.538 |
| **Pleurisy** | 11 (2.34%) | 24 (1.27%) | 13 (0.55%) | 1.000 |
| **Proteinuria** | 81 (17.20%) | 202 (10.70%) | 0 (0.00%) | <0.001* |
| **Psychosis** | 220 (46.71%) | 149 (7.90%) | 1 (0.04%) | <0.001* |
| **Pyuria** | 264 (56.05%) | 455 (24.11%) | 11 (0.47%) | <0.001* |
| **Rash** | 150 (31.85%) | 443 (23.48%) | 165 (6.99%) | 0.200 |
| **Urinary casts** | 21 (4.46%) | 56 (2.97%) | 20 (0.85%) | <0.001* |
| **Seizure** | 125 (26.54%) | 83 (4.40%) | 1 (0.04%) | <0.001* |
| **Thrombocytopenia** | 71 (15.07%) | 146 (7.74%) | 83 (3.52%) | 0.702 |
| **Vasculitis** | 8 (1.70%) | 15 (0.79%) | 4 (0.17%) | 1.000 |
| **Visual disturbance** | 54 (11.46%) | 111 (5.88%) | 65 (2.76%) | 0.002* |

* Statistical significance

**Table S3: Performance metrics for XGboost and Logistic Regression**

| Random Over Sampling |  |  | Full variable 3 Months | Clinical 3 Months | SDoH 3 Months |
| --- | --- | --- | --- | --- | --- |
|  | F1- Score | Xgboost | 0.35 | 0.36 | 0.27 |
|  |  | Logistic Regression | 0.33 | 0.34 | 0.28 |
|  | AUROC | Xgboost | 0.66 | 0.67 | 0.54 |
|  |  | Logistic Regression | 0.65 | 0.67 | 0.55 |
|  | Recall | Xgboost | 0.56 | 0.67 | 0.59 |
|  |  | Logistic Regression | 0.48 | 0.52 | 0.56 |
|  | Precision | Xgboost | 0.26 | 0.25 | 0.18 |
|  |  | Logistic Regression | 0.25 | 0.26 | 0.19 |
|  | Specificity | Xgboost | 0.69 | 0.61 | 0.48 |
|  |  | Logistic Regression | 0.72 | 0.71 | 0.53 |
| Random Under Sampling |  |  | Full variable 3 Months | Clinical 3 Months | SDoH 3 Months |
|  | F1- Score | Xgboost | 0.35 | 0.36 | 0.26 |
|  |  | Logistic Regression | 0.34 | 0.34 | 0.27 |
|  | AUROC | Xgboost | 0.67 | 0.67 | 0.55 |
|  |  | Logistic Regression | 0.65 | 0.67 | 0.56 |
|  | Recall | Xgboost | 0.60 | 0.63 | 0.47 |
|  |  | Logistic Regression | 0.51 | 0.51 | 0.54 |
|  | Precision | Xgboost | 0.25 | 0.25 | 0.18 |
|  |  | Logistic Regression | 0.25 | 0.26 | 0.18 |
|  | Specificity | Xgboost | 0.66 | 0.64 | 0.59 |
|  |  | Logistic Regression | 0.71 | 0.72 | 0.53 |

**Table S4: Distribution of Top 10 clinical variables in XGBoost ROS SHAP analysis**

|  | -12 ~ -10 months | -9 ~ -7 months | -6 ~ -4 months | -3 ~ -1 months | +1 ~ +3 months |
| --- | --- | --- | --- | --- | --- |
| HEADACHE | 1798 | 2189 | 2644 | 2589 | 2546 |
| ORGANICBRAIN | 1149 | 1481 | 1862 | 1844 | 1716 |
| PYURIA | 933 | 1158 | 1378 | 1451 | 1464 |
| PROTEINURIA | 443 | 600 | 714 | 675 | 594 |
| RASH | 1434 | 1750 | 2111 | 2057 | 1904 |
| PSYCHOSIS | 949 | 1160 | 1453 | 1456 | 1375 |
| FEVER | 415 | 533 | 652 | 670 | 621 |
| SEIZURE | 612 | 752 | 892 | 917 | 896 |
| CVA | 362 | 466 | 600 | 592 | 569 |
| HEMATURIA | 341 | 430 | 493 | 495 | 482 |

**Figure S1: Performance assessment by AUROC for the logistic regression model**


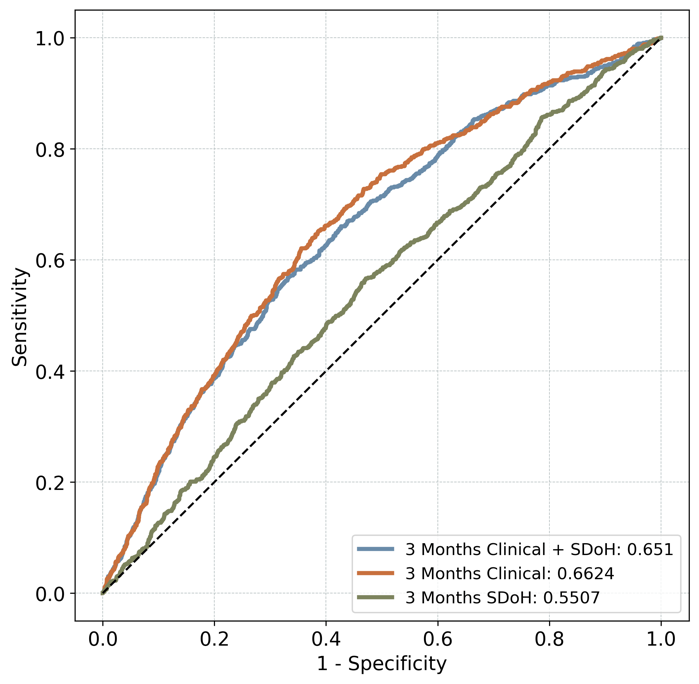


**Figure S2: Flare risk by FLAME decile using logistic regression on independent data**


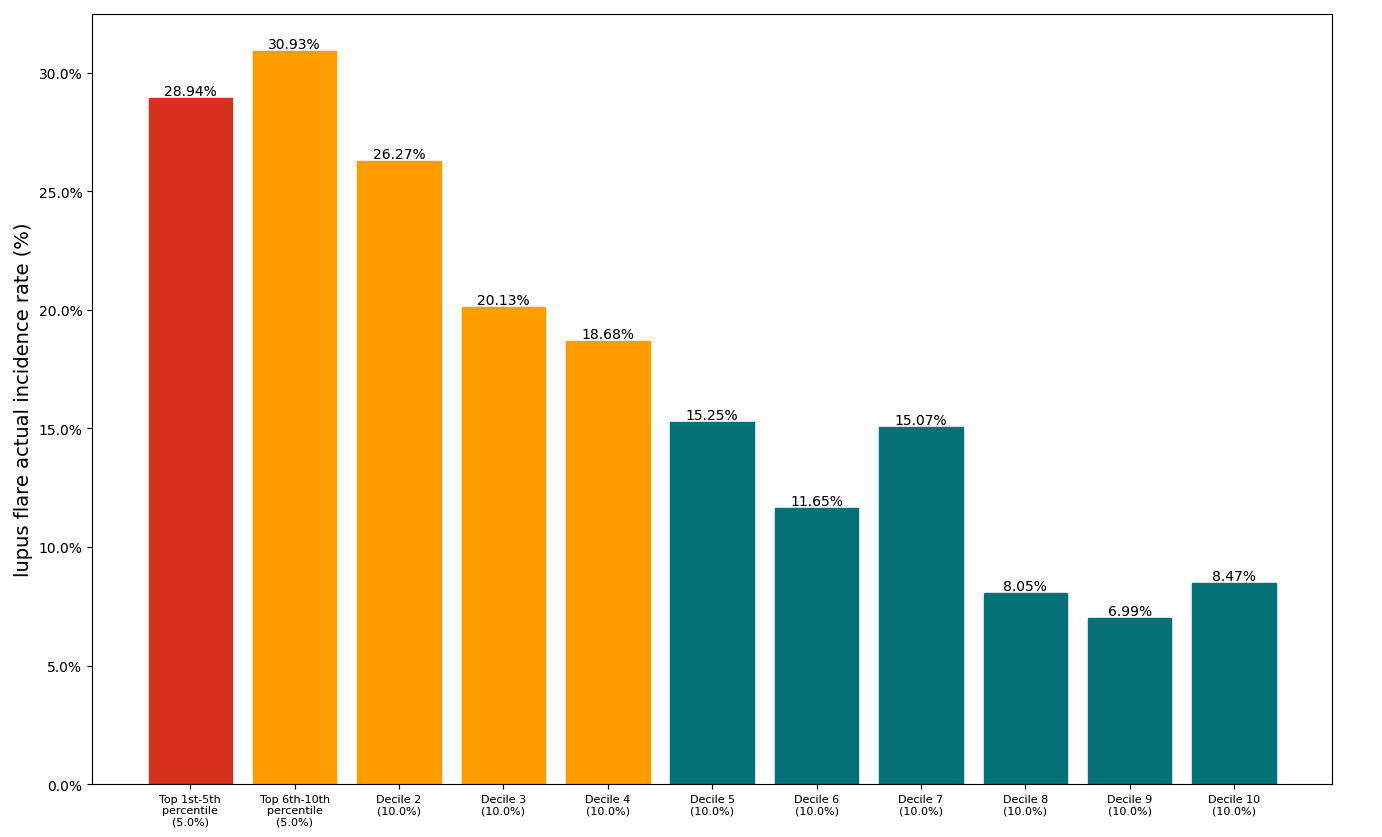


**Figure S3: Feature importance analysis with SHAP values (Logistic regression model)**


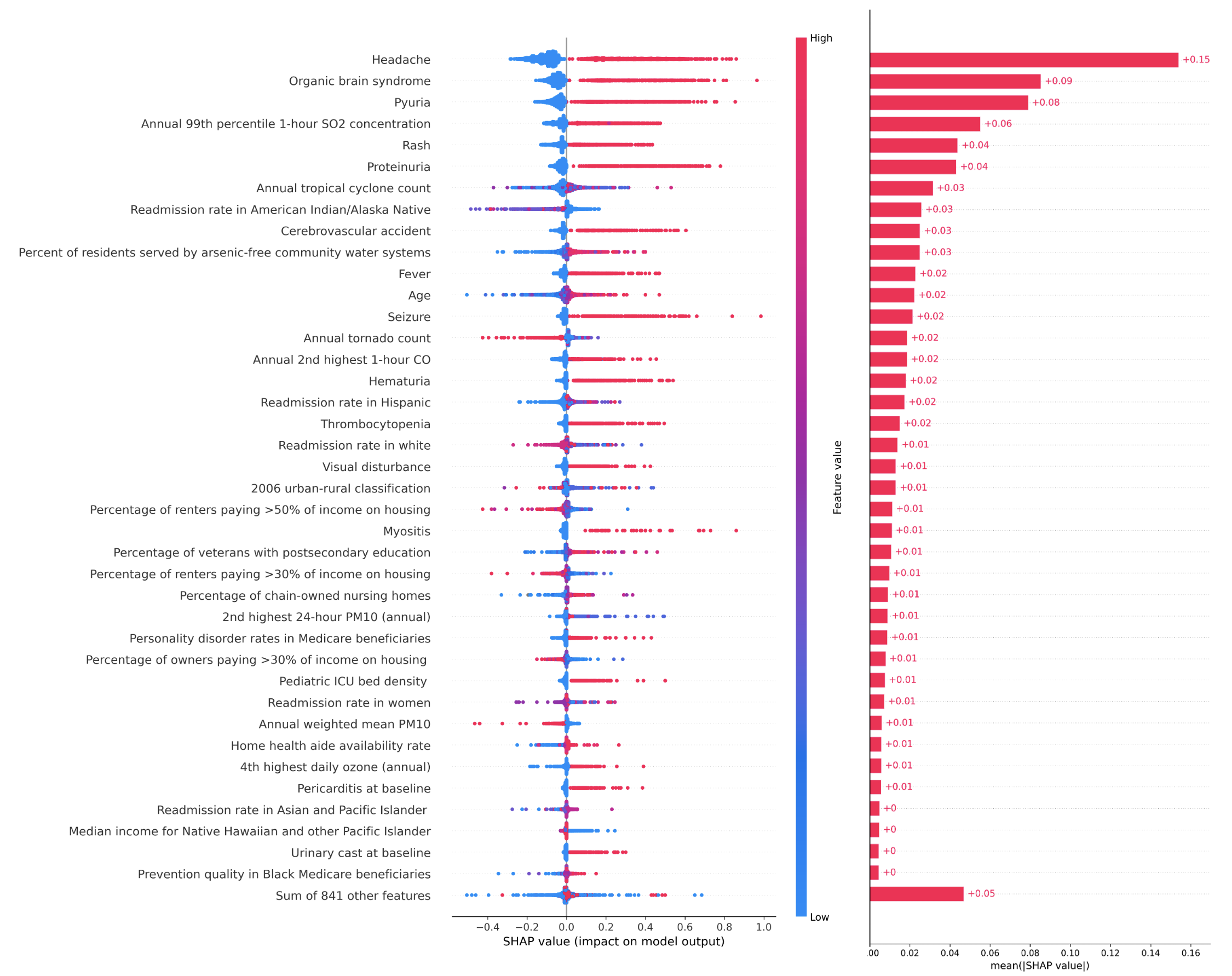


**Figure S4: False negative rate (FNR) curve across different population groups using Xgboost**


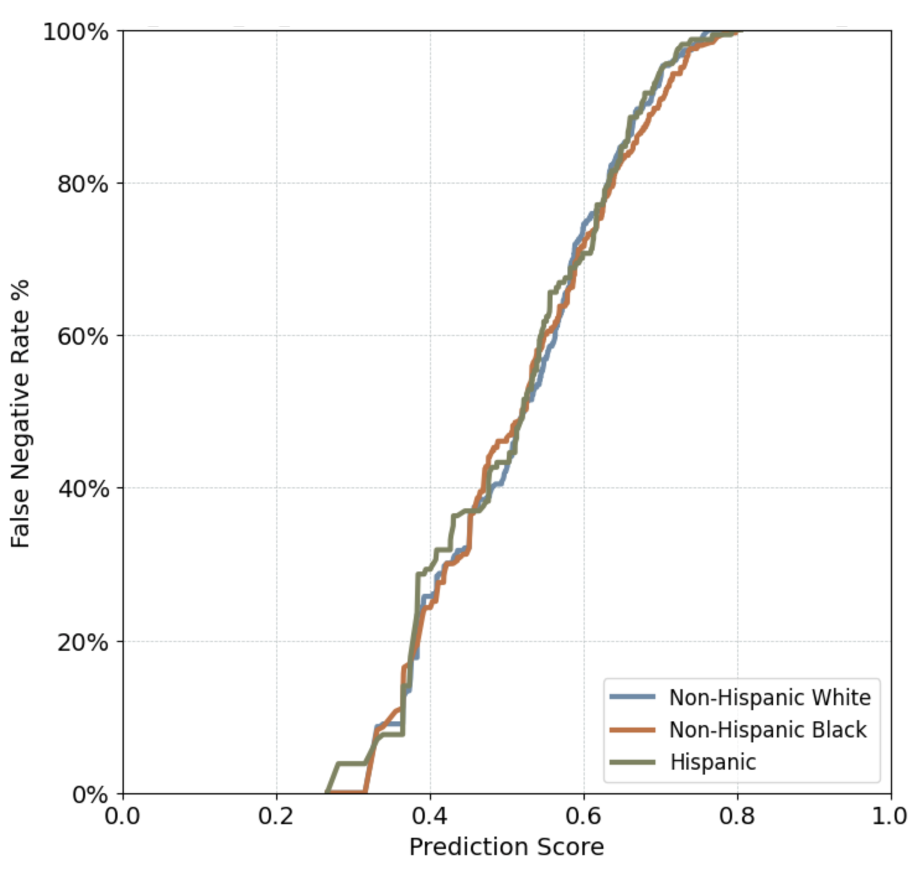


**Figure S5: False negative rate (FNR) curve between different populations using logistic regression**


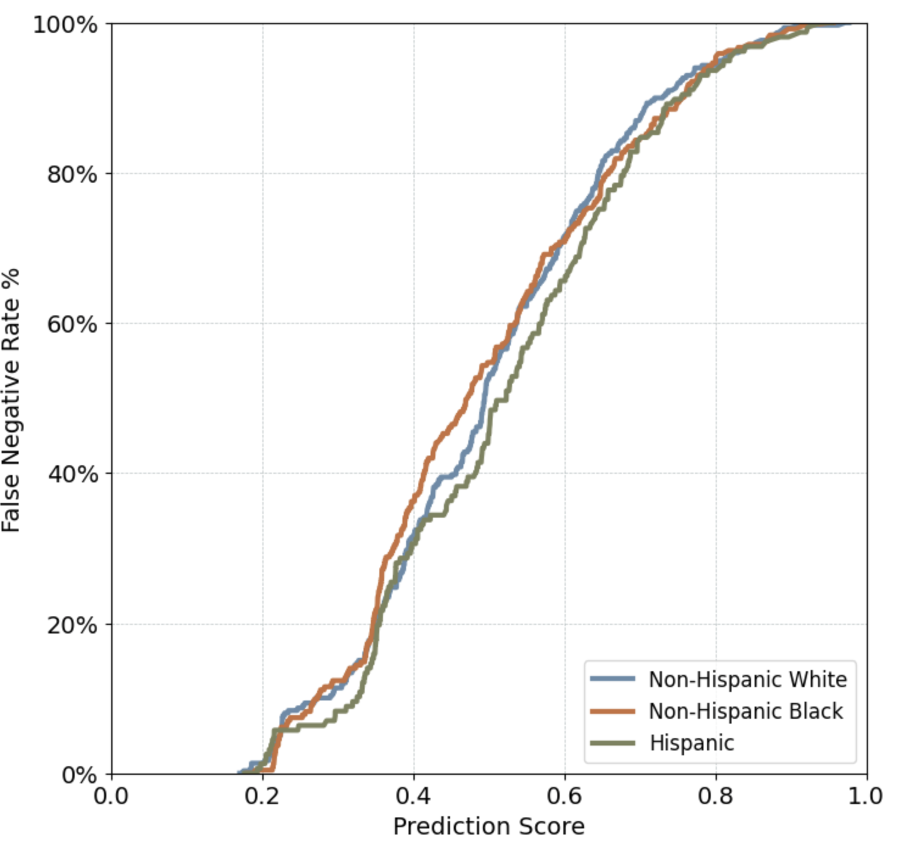


**Reference**

1. Shin J, Lee J, Ko T, Lee K, Choi Y, Kim HS. Improving Machine Learning Diabetes Prediction Models for the Utmost Clinical Effectiveness. *J Pers Med*. 2022;12(11). doi:10.3390/jpm12111899

2. Zhao Y, Li X, Li S, et al. Using Machine Learning Techniques to Develop Risk Prediction Models for the Risk of Incident Diabetic Retinopathy Among Patients With Type 2 Diabetes Mellitus: A Cohort Study. *Front Endocrinol* . 2022;13:876559.

3. Deberneh HM, Kim I. Prediction of Type 2 Diabetes Based on Machine Learning Algorithm. *Int J Environ Res Public Health*. 2021;18(6). doi:10.3390/ijerph18063317

4. Li Y, Wang H, Luo Y. Improving Fairness in the Prediction of Heart Failure Length of Stay and Mortality by Integrating Social Determinants of Health. *Circ Heart Fail*. 2022;15(11):e009473.

5. Yang H, Li J, Liu S, Yang X, Liu J. Predicting Risk of Hypoglycemia in Patients With Type 2 Diabetes by Electronic Health Record-Based Machine Learning: Development and Validation. *JMIR Med Inform*. 2022;10(6):e36958.

6. Wang L, Wang X, Chen A, Jin X, Che H. Prediction of Type 2 Diabetes Risk and Its Effect Evaluation Based on the XGBoost Model. *Healthcare (Basel)*. 2020;8(3). doi:10.3390/healthcare8030247

7. Tolles J, Meurer WJ. Logistic Regression: Relating Patient Characteristics to Outcomes. *JAMA*. 2016;316(5):533-534.

8. Lundberg S, Lee SI. A unified approach to interpreting model predictions. *arXiv [csAI]*. Published online May 22, 2017. Accessed January 15, 2023. https://proceedings.neurips.cc/paper/2017/hash/8a20a8621978632d76c43dfd28b67767-Abstract.html

9. Lee JD, Hastie TJ. Learning the Structure of Mixed Graphical Models. *J Comput Graph Stat*. 2015;24(1):230-253.

10. Raghu VK, Poon A, Benos PV. Evaluation of Causal Structure Learning Methods on Mixed Data Types. *Proc Mach Learn Res*. 2018;92:48-65.

11. Colombo D, Maathuis MH. Order-independent constraint-based causal structure learning. *J Mach Learn Res*. Published online 2014. https://www.jmlr.org/papers/volume15/colombo14a/colombo14a.pdf

12. Spirtes P, Glymour C, Scheines R. *Causation, Prediction, and Search*. Springer New York

13. Patient flow with Sankey diagrams or Circos plots. Accessed February 25, 2025. https://sebastiz.github.io/gastrodatascience/DataAnalysisEventingsSankey.html

14. Accessed April 5, 2024. https://www.sciencedirect.com/science/article/pii/S1521694223000803#:~:text=A

15. Chouldechova A. Fair prediction with disparate impact: A study of bias in recidivism prediction instruments. *Big Data*. 2017;5(2):153-163.
